# Comprehensive Evaluation of YJ‐2 as a PAD4 Inhibitor in Alleviating Ischemic Brain Injury: From NETs‐Induced Neurotoxicity to In Vivo Neuroprotection

**DOI:** 10.1002/cns.71032

**Published:** 2026-07-15

**Authors:** Yijiang Jia, Ayijiang Taledaohan, Kai Wang, Maer Maer Tuohan, Rong Chen, Liujia Chan, Haoyu Shen, Di Zhu, Yuji Wang

**Affiliations:** ^1^ Department of Medicinal Chemistry School of Pharmaceutical Sciences of Capital Medical University Beijing China; ^2^ Beijing Key Laboratory of Drug Innovation for Neuro‐Oncology, Beijing Engineering Research Center of Targeted Drugs and Cell Therapy for CNS Tumors, Laboratory for Clinical Medicine Capital Medical University Beijing China; ^3^ Department of Emergency Medicine University of California San Diego San Diego California USA; ^4^ Department of Clinical Pharmacy The First Affiliated Hospital of Shandong First Medical University and Shandong Provincial Qianfoshan Hospital Jinan Shandong China; ^5^ Department of Pharmacy, Xuanwu Hospital Capital Medical University Beijing China

**Keywords:** blood–brain barrier, ischemia/reperfusion injury, neuroprotection, neutrophil extracellular traps (NETs), PAD4 inhibitor

## Abstract

**Aims:**

To evaluate the neuroprotective potential of YJ‐2, a novel peptidylarginine deiminase 4 (PAD4) inhibitor, against ischemia/reperfusion brain injury by targeting neutrophil extracellular trap (NET) formation.

**Methods:**

In vitro, a NETs‐induced injury model was established using SH‐SY5Y and bEnd.3 cells. YJ‐2's effects on viability, apoptosis, oxidative stress, and barrier permeability were assessed via CCK‐8, flow cytometry, and FITC‐dextran assays. In vivo, a rat middle cerebral artery occlusion/reperfusion (MCAO/R) model received YJ‐2 (10 μmol/kg) intravenously. Outcomes included infarct volume (TTC staining), neurological score, neuronal apoptosis (TUNEL), and oxidative markers (ELISA). PAD4 activity and histone H3 citrullination (H3cit) were examined by western blot and immunofluorescence.

**Results:**

YJ‐2 reduced NET‐mediated neuronal death and oxidative stress in vitro, and improved endothelial barrier integrity. In MCAO/R rats, YJ‐2 significantly lowered infarct volume (44.2% → 30.6%), improved neurological function, and suppressed apoptosis. It also decreased PAD4 and H3cit expression in ischemic brain tissue, confirming target engagement.

**Conclusion:**

YJ‐2, by preserving blood–brain barrier (BBB) integrity and reducing neuronal apoptosis, highlights its therapeutic potential for ischemic stroke.

## Graphical abstract

The novel PAD4 inhibitor YJ‐2 potently suppresses neutrophil extracellular trap (NETs) formation. In a rat model of ischemic stroke, YJ‐2 treatment significantly reduces cerebral infarct volume and improves neurological function, demonstrating its therapeutic potential.

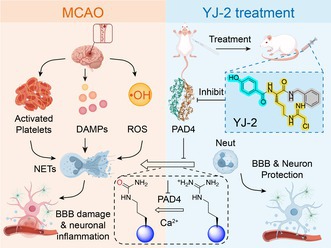

## 1 Introduction

Ischemic stroke represents a leading global cause of mortality and long‐term disability. According to the Global Burden of Disease Study 2019, stroke was the second‐leading cause of death worldwide, resulting in approximately 6.55 million deaths annually, with ischemic stroke constituting 62.4% of all incident cases [1, 2]. This staggering burden underscores the urgent need for effective therapeutic strategies. The cornerstone of acute management relies on timely reperfusion, primarily achieved through intravenous thrombolysis with recombinant tissue plasminogen activator (t‐PA) or endovascular thrombectomy (EVT) [3, 4, 5]. While these interventions are crucial for restoring blood flow, the process of reperfusion itself can paradoxically induce additional brain injury, known as ischemia/reperfusion (I/R) injury, which significantly contributes to the final neurological deficit [6, 7]. This secondary injury phase is characterized by a complex cascade involving inflammatory responses, oxidative stress, and cellular death [8, 9].

Within this cascade, neutrophil‐mediated inflammation plays a pivotal role in exacerbating brain injury [10, 11, 12, 13]. A key effector mechanism is the formation of neutrophil extracellular traps (NETs)—web‐like structures composed of decondensed DNA, histones, and granular enzymes released by activated neutrophils. In ischemic stroke, NETs promote stable, lysis‐resistant thrombus formation, directly compromise blood–brain barrier (BBB) integrity, and amplify neuroinflammation, thereby fueling a vicious cycle of secondary brain damage [14, 15, 16, 17].

The formation of NETs is critically dependent on the enzyme peptidylarginine deiminase 4 (PAD4), which catalyzes histone citrullination, leading to chromatin decondensation and NET release [12, 18]. Consequently, pharmacological inhibition of PAD4 has emerged as a promising strategy to mitigate NETs‐induced injury [19, 20]. First‐generation PAD4 inhibitors, such as Cl‐amidine, have validated this therapeutic concept in preclinical models, demonstrating attenuation of vascular damage and reduced immune cell infiltration when NETosis is suppressed in middle cerebral artery occlusion (MCAO) animals [15, 21]. However, their moderate inhibitory potency (e.g., Cl‐amidine IC_50_ = 5.9 μM) limits standalone efficacy, often necessitating advanced delivery strategies to achieve sufficient brain exposure [22, 23]. For example, to overcome poor pharmacokinetics, Cl‐amidine has been incorporated into engineered nanocarriers, such as ROS‐responsive and fibrin‐targeted liposomes, to enhance its accumulation and activity at ischemic sites [24]. Similarly, structurally optimized drugs like intranasally administered BB‐Cl‐amidine demonstrated efficacy in MCAO models, significantly reducing infarct volume and promoting functional recovery [25]. Nevertheless, these approaches underscore a fundamental limitation: the reliance on complex delivery systems to compensate for the drug's suboptimal plasma pharmacokinetics and brain penetration.

To address these inherent pharmacokinetic challenges, we rationally designed the novel PAD4 inhibitor YJ‐2 through strategic structural optimization, aiming to enhance target potency and central nervous system accessibility. This design incorporates a benzylamino group to strengthen hydrophobic interactions within the enzyme pocket, while a 4‐hydroxybenzamide terminus provides critical hydrogen bonding and optimizes molecular polarity. These modifications resulted in a compound with markedly enhanced in vitro potency, exhibiting an IC_50_ of 0.836 ± 0.240 μM against PAD4—approximately sevenfold more potent than Cl‐amidine and superior to BB‐Cl‐amidine [22, 26]. In this study, we comprehensively evaluated YJ‐2 and hypothesized that its enhanced activity would translate into robust neuroprotection by effectively disrupting the PAD4‐NETs axis. To test this hypothesis, we conducted integrated in vitro and in vivo investigations to: (1) characterize its protective effects against NETs‐induced neuronal cytotoxicity and BBB damage; (2) evaluate its efficacy in improving histological and functional outcomes in a rat model of cerebral ischemia–reperfusion injury; and (3) validate its target engagement and brain penetration. Our work establishes YJ‐2 as a next‐generation therapeutic candidate that combines high intrinsic potency with a favorable profile for potential systemic administration in the treatment of ischemic stroke.

## 2 Materials and Methods

### 2.1 Materials

The mouse brain microvascular endothelial cell line (bEnd.3) and human neuroblastoma cells (SH‐SY5Y) were obtained from the American Type Culture Collection (ATCC). Edaravone (EDA) (Sigma‐Aldrich), cell culture media and reagents (KeyGEN, Gibco), fluorescein isothiocyanate‐dextran (FITC‐dextran), 2′,7′‐dichlorofluorescin diacetate (DCFH‐DA), Cell Counting Kit‐8 (CCK‐8), Annexin V‐FITC Apoptosis Kit, and ELISA kits for glutathione peroxidase (GSH‐Px) and malondialdehyde (MDA) were used. Primary antibodies against H3cit, PADI4, GAPDH, and secondary antibodies were from Abcam.

### 2.2 Synthesis of YJ‐2

The compound YJ‐2 (chemical structure shown in Figure S1) was prepared as described previously [26]. Please refer to the cited reference for detailed synthesis and characterization.

### 2.3 Statistical Analysis

Data were presented as the mean ± standard deviation (SD) from at least three independent experiments. Normality and homoscedasticity were verified using Shapiro–Wilk and Levene's tests, respectively, prior to one‐way ANOVA (GraphPad Prism 9.5.1). Statistical significance was defined as *p < 0.05 and **p < 0.01.

### 2.4 Isolation of Neutrophils and Establishment of a NETs Co‐Culture Model

Mouse bone marrow neutrophils were isolated via Percoll density gradient centrifugation [16, 20]. Cells (5 × 10^5^/well) were pretreated with YJ‐2 or vehicle for 30 min, then stimulated with 100 nM phorbol 12‐myristate 13‐acetate (PMA) for 4 h to induce NETosis. The supernatant containing NETs components was collected and applied to SH‐SY5Y cells for 24 h to establish the injury model.

### 2.5 Neuroprotective Effect of YJ‐2 In Vitro

To evaluate the neuroprotective effect of YJ‐2, SH‐SY5Y cell viability was measured using the CCK‐8 assay. Cells were seeded in 96‐well plates (1 × 10^4^ cells/well) for 24 h. The culture medium was then replaced with supernatant from PMA‐stimulated neutrophils that had been pre‐incubated with different concentrations of YJ‐2 for 24 h. After this treatment, 10 μL of CCK‐8 reagent was added to each well, followed by a 2 h incubation. The absorbance at 450 nm was measured using a microplate reader.

### 2.6 Flow Cytometric Analysis of Apoptosis and ROS

SH‐SY5Y cells treated for 24 h with neutrophil supernatants were analyzed by flow cytometry for apoptosis (Annexin V‐FITC/PI kit) and intracellular ROS (DCFH‐DA), per manufacturer protocols. The experiment included the following groups: (A) Control supernatant group; (B) PMA‐stimulated neutrophil supernatant group; and (C) YJ‐2 (50 μM) + PMA‐stimulated neutrophil supernatant group.

### 2.7 In Vitro BBB Protection Assay

An in vitro BBB model was established using the bEnd.3 cells. bEnd.3 cells were cultured on Transwell inserts to form a confluent monolayer. After pretreatment with YJ‐2 for 2 h, the monolayers were exposed to 50% (v/v) NETs supernatant for 24 h. Barrier permeability was assessed using FITC‐dextran (10 kDa); fluorescence in the lower chamber was measured after 2 h.

### 2.8 The Middle Cerebral Artery Ischemia (MCAO) Model

Male Sprague–Dawley rats (weighing 250–280 g) were subjected to transient MCAO as previously described [27], with minor modifications. Briefly, under isoflurane anesthesia, a silicone‐coated filament was inserted via the external carotid artery (ECA) to occlude the middle cerebral artery for 2 h, followed by reperfusion. Sham rats underwent surgery without occlusion. Post‐reperfusion, rats received tail vein injections of normal saline (NS), EDA (3 mg/kg), or YJ‐2 (10 μmol/kg).

### 2.9 Neurological Deficit Assessment

Neurological deficits were evaluated at 2 h after MCAO and again at 22 h post‐reperfusion using the ZeLonga scoring system. A researcher who was blinded to the experimental groups performed all assessments. A higher score indicates more severe neurological impairment.

### 2.10 Measurement of Cerebral Infarct Volume by TTC Staining

At 22 h post‐reperfusion, brains were sectioned and stained with 2% 2,3,5‐triphenyltetrazolium chloride (TTC) at 37°C for 20 min. Infarct volume was analyzed from photographed sections using ImageJ.

### 2.11 Histological Staining (TUNEL, Nissl, and H&E)

Paraffin‐embedded brain sections (5 μm) were used. TUNEL staining was performed per kit instructions (Servicebio) to detect apoptotic cells. For Nissl and H&E staining, sections were deparaffinized, rehydrated, and stained with toluidine blue or hematoxylin/eosin, respectively, following standard protocols. Stained sections were imaged under a light microscope.

### 2.12 Measurement of Serum Oxidative Stress Markers by ELISA

Serum samples were obtained from whole blood collected under isoflurane anesthesia via orbital puncture and subsequent centrifugation at 3500 rpm for 10 min. The concentrations of oxidative stress markers, including GSH‐Px, ROS, and MDA, were quantified using respective commercial ELISA kits in strict accordance with the manufacturers' instructions.

### 2.13 Western Blot Analysis

Protein from ischemic cortex was extracted, separated by SDS‐PAGE, and transferred to PVDF membranes. After blocking, membranes were incubated overnight with primary antibodies (H3cit, PADI4, GAPDH), followed by HRP‐conjugated secondary antibody. Bands were visualized by ECL and quantified with ImageJ.

### 2.14 Immunofluorescence Staining

Brain sections were processed for antigen retrieval, blocked, and incubated overnight with anti‐H3cit antibody. After washing, sections were incubated with FITC‐conjugated secondary antibody, counterstained with DAPI, and imaged by confocal microscopy.

## 3 Results

### 3.1 Neuroprotective Effects of YJ‐2 In Vitro

**FIGURE 1:**
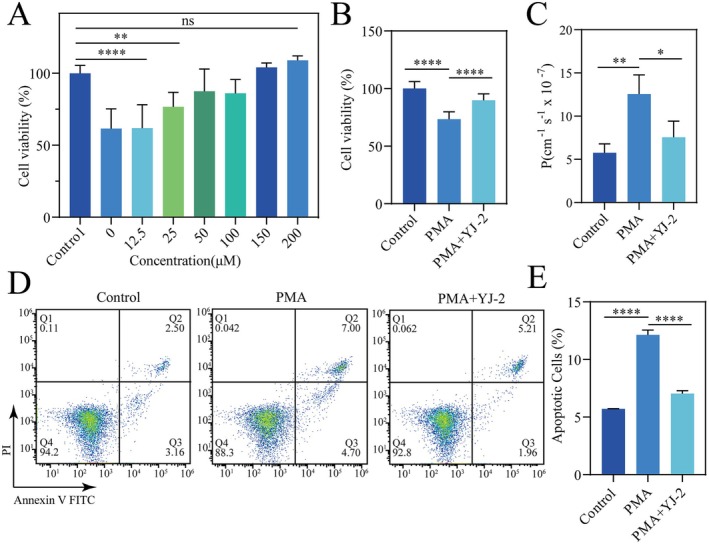
In vitro neuroprotective effects of YJ‐2. (A) Viability of SH‐SY5Y cells treated with supernatant from unstimulated neutrophils in the presence of increasing concentrations of YJ‐2 (0–200 μM) for 24 h, as determined by CCK‐8 assay (*n* = 6). (B) Viability of SH‐SY5Y cells treated for 24 h with supernatants from neutrophils under three conditions: unstimulated (Control), PMA‐stimulated (PMA), and PMA‐stimulated with YJ‐2 pretreatment (50 μM, PMA + YJ‐2) (*n* = 6). (C) Permeability coefficient (P) of endothelial monolayers of bEnd.3 cells using 10 kDa FITC‐dextran as probe at 2 h treated by supernatant of PMA stimulated neutrophil with different formulations (*n* = 4). (D) Analysis of apoptosis and necrosis in SH‐SY5Y cells under different treatment conditions by Annexin V‐FITC/PI double‐staining flow cytometry. (E) Statistical analysis of the apoptosis rates for each group (*n* = 6). Data are presented as mean ± SD; ^ns^ *p* > 0.05, *p < 0.05, **p < 0.01, ****p < 0.0001.

To evaluate the potential protective effects of YJ‐2 against NETs‐mediated damage, SH‐SY5Y cells were co‐treated with supernatant from unstimulated neutrophils and increasing concentrations of YJ‐2 (0–200 μM) for 24 h, followed by CCK‐8 viability assay. As shown in Figure 1A, cells treated with neutrophil supernatant in the absence of YJ‐2 (0 μM) exhibited a significant reduction in viability compared to the control (*p* < 0.0001), confirming the basal cytotoxic effect of neutrophil‐derived components. YJ‐2 reversed this cytotoxicity in a concentration‐dependent manner. At concentrations of 50 μM and above, cell viability was restored to a level not significantly different from that of the control group, indicating that this concentration range affords complete protection. Therefore, 50 μM was selected as the working concentration for all subsequent in vitro studies investigating the neuroprotective mechanism of YJ‐2. Based on this, treatment with supernatant from PMA‐activated neutrophils significantly reduced the viability of SH‐SY5Y cells, decreasing the survival rate to 76.2% (*p* < 0.0001, vs. control). However, pretreatment with YJ‐2 markedly reversed this NETs‐mediated cellular injury, restoring the survival rate to 89.81% (compared to the PMA group, *p* < 0.0001), thereby confirming the definite protective effect of YJ‐2 against NETs‐induced neuronal damage in vitro (Figure 1B).

### 3.2 YJ‐2 Reduces NETs Supernatant‐Induced Apoptosis in SH‐SY5Y Cells

To elucidate the protective mechanism of YJ‐2 against cell damage caused by NETs supernatant, we precisely analyzed the mode of SH‐SY5Y cell death using Annexin V‐FITC/PI double‐staining flow cytometry. As shown in Figure 1D, compared to the normal control group, treatment with NETs supernatant significantly increased the proportion of Annexin V‐positive cells. Further statistical analysis (Figure 1E) revealed that NETs supernatant markedly elevated the percentage of apoptotic cells (Annexin V^+^) from 5.70% in the control group to 12.14% (*p* < 0.0001). Notably, pretreatment with YJ‐2 significantly attenuated this NETs‐induced apoptosis, reducing the apoptotic cell proportion to 7.05% (*p* < 0.0001). These results clearly demonstrate that YJ‐2 effectively mitigates NETs supernatant‐induced apoptotic cell death, thereby contributing to the maintenance of cell survival.

### 3.3 YJ‐2 Attenuates NETs Supernatant‐Induced Oxidative Stress

**FIGURE 2:**
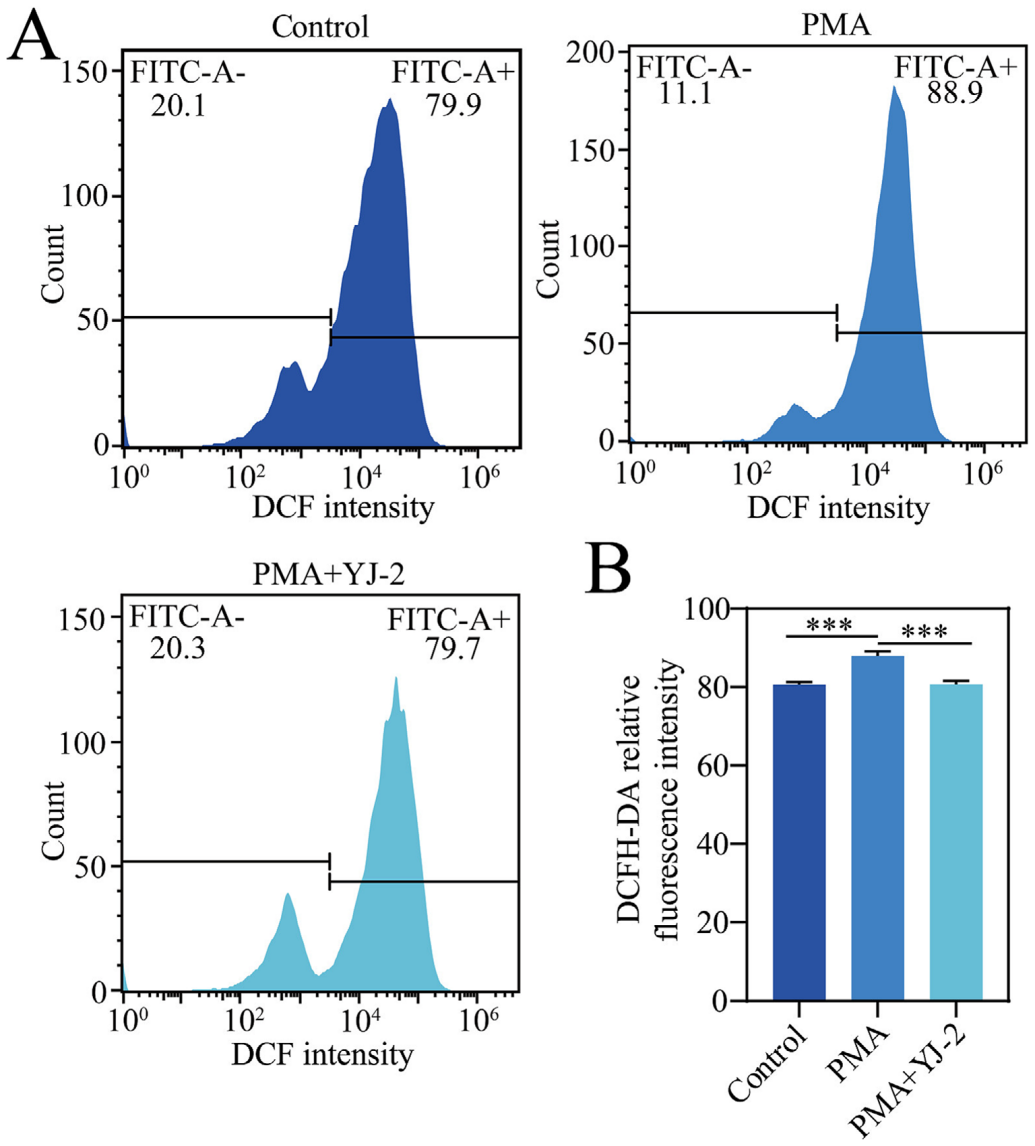
YJ‐2 alleviates oxidative stress to exert neuroprotective effects in vitro. (A) Representative flow cytometry histograms of DCFH‐DA‐stained SH‐SY5Y cells, showing intracellular ROS levels under Control, PMA‐stimulated NETs supernatant, and YJ‐2 + PMA treatment conditions (*n* = 3). (B) Quantitative analysis of the percentage of ROS‐positive cells across treatment groups. All data are presented as mean ± SD. ***p < 0.001.

To investigate whether the neuroprotective effect of YJ‐2 involves the alleviation of oxidative stress, intracellular reactive oxygen species (ROS) levels were measured using the DCFH‐DA fluorescent probe coupled with flow cytometry. As shown in Figure 2A,B, stimulation with NETs supernatant resulted in a sharp increase in intracellular ROS levels in SH‐SY5Y cells compared to the control group (*p* < 0.001), confirming that NETs components trigger a potent oxidative stress response. However, intervention with YJ‐2 significantly reversed this effect. Pretreatment with YJ‐2 markedly reduced the intracellular ROS fluorescence intensity compared to the PMA group (*p* < 0.001), restoring it to a level comparable to the Control group. This finding strongly demonstrates that the protective role of YJ‐2 is closely associated with its effective mitigation of intracellular oxidative stress, suggesting that it may either directly scavenge ROS or enhance the cell's intrinsic antioxidant defense capacity, thereby blocking the cellular damage pathways activated by oxidative stress.

### 3.4 YJ‐2 Alleviates NETs‐Induced BBB Damage in an In Vitro Model

Disruption of the BBB in the early phase of ischemic stroke is a critical event that facilitates the infiltration of immune cells, such as neutrophils, and exacerbates neurological injury. Studies suggest that NETs may further compromise BBB integrity by damaging the basement membrane and vascular structure [28, 29]. To evaluate the protective potential of YJ‐2 against stroke‐associated BBB injury, an in vitro BBB model was established using bEnd.3 cells in a Transwell system, and barrier integrity was assessed via a FITC‐dextran permeability assay. Results demonstrated that treatment with NETs supernatant significantly impaired the integrity of the bEnd.3 monolayer, as reflected by a 66.8% increase in the permeability coefficient (*P*) compared to the Control group (Figure 1C). In contrast, intervention with YJ‐2 markedly improved monolayer integrity, reducing the permeability coefficient by 21.3% compared to the NETs model group. These findings indicate that YJ‐2 effectively attenuates NETs‐induced BBB damage, demonstrating its endothelial protective role in this in vitro model.

### 3.5 YJ‐2 Reduces Cerebral Infarct Volume and Neuronal Apoptosis After Cerebral Ischemia–Reperfusion Injury

**FIGURE 3:**
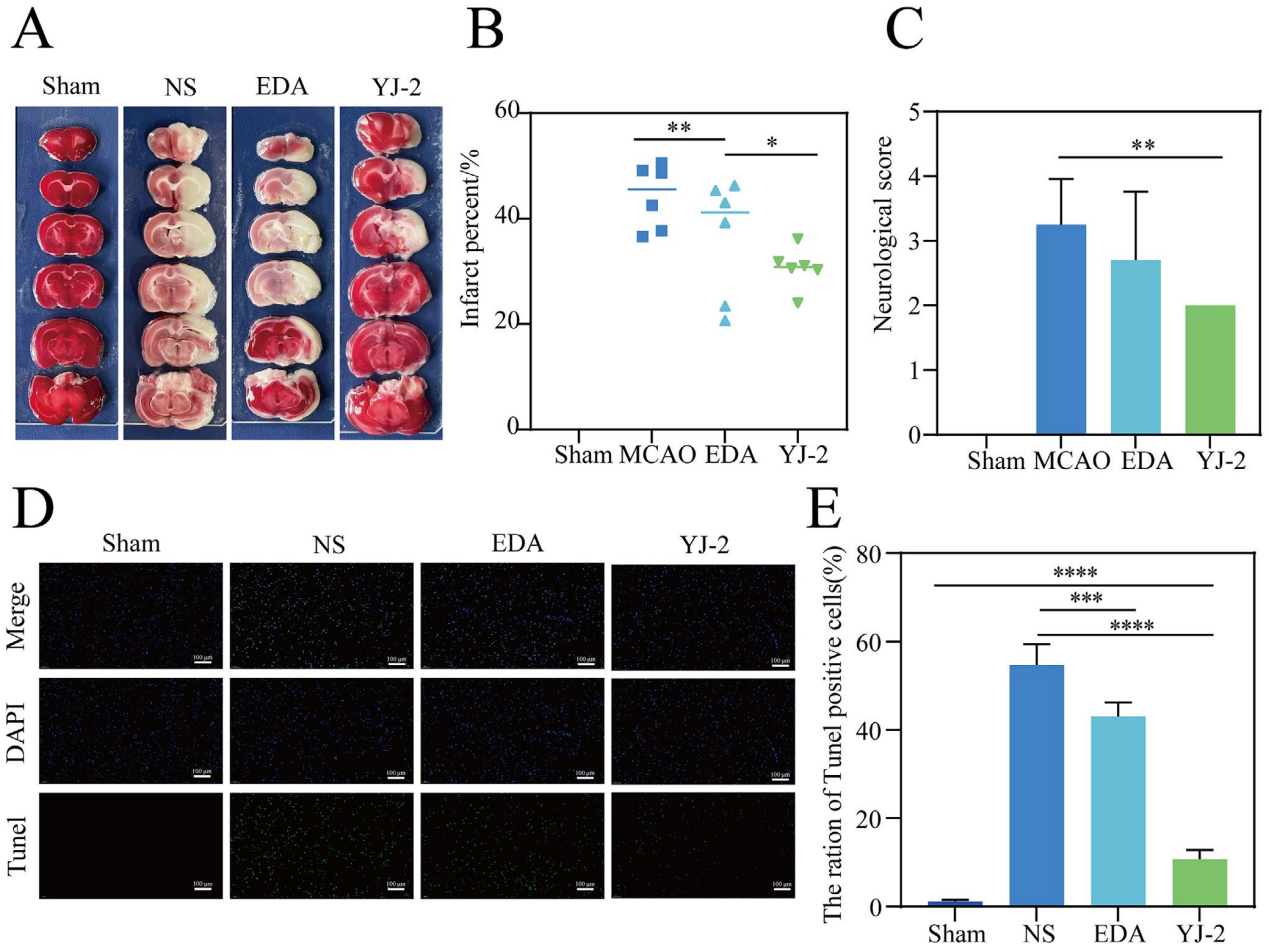
Neuroprotective effects of YJ‐2 against cerebral ischemia–reperfusion injury. (A) Representative images of TTC‐stained rat brain coronal sections from Sham, MCAO/*R* + NS (Model), MCAO/*R* + EDA (3 mg/kg, positive control), and MCAO/*R* + YJ‐2 (10 μmol/kg) groups at 22 h after reperfusion. Viable tissue appears red, while the infarct area is white. (B) Statistical analysis of cerebral infarct volume (*n* = 6). (C) Neurological deficit scores (modified Neurological Severity Score, mNSS) (*n* = 6). Higher scores indicate more severe neurological impairment. (D) Representative TUNEL staining images of the ischemic penumbra from each group. Apoptotic cells are labeled with green fluorescence (scale bar: 100 μm). (E) Statistical analysis of the percentage of TUNEL‐positive cells (*n* = 6). Data are expressed as mean ± SD. *p < 0.05, **p < 0.01, ***p < 0.001, ****p < 0.0001.

To systematically evaluate the neuroprotective efficacy of YJ‐2 in vivo, we employed a rat model of MCAO/R and conducted multidimensional analyses encompassing histopathology, neurological function, and cellular apoptosis. TTC staining results (Figure 3A,B) revealed extensive cerebral infarction in the model (NS) group at 22 h post‐reperfusion, with an infarct volume of 44.2%, primarily involving the cortical and striatal regions, indicating successful establishment of the cerebral ischemia model. Administration of YJ‐2 significantly reduced the infarct area, decreasing the volume to 30.6%. This outcome showed statistically significant differences compared to both the NS group and the positive control EDA group (*p* < 0.05), suggesting that YJ‐2 effectively limits the expansion of cerebral tissue damage during the ischemia–reperfusion process.

Neurological deficit scores further validated the therapeutic potential of YJ‐2 (Figure 3C). Rats in the MCAO model group exhibited severe motor and balance dysfunction with significantly elevated scores, whereas the YJ‐2 treatment group showed markedly improved neurobehavioral performance and significantly reduced scores. This demonstrates that YJ‐2 alleviates I/R‐induced neurological impairment at the functional level.

To further investigate the effect of YJ‐2 on neuronal survival, TUNEL staining was performed to detect cellular apoptosis in the ischemic penumbra. Figure 3E demonstrates a significant increase in TUNEL‐positive cells in the model group, indicating extensive neuronal apoptosis induced by I/R. In contrast, the YJ‐2 treatment group exhibited a significantly lower proportion of TUNEL‐positive cells, suggesting that YJ‐2 inhibits the I/R‐activated apoptotic pathway, thereby exerting a neuroprotective effect. Collectively, these in vivo findings demonstrate that YJ‐2 provides significant neuroprotection by reducing cerebral infarct volume, improving neurological function, and suppressing neuronal apoptosis, highlighting its multi‐faceted therapeutic potential.

### 3.6 YJ‐2 Suppresses Oxidative Stress Following MCAO/R

**FIGURE 4:**
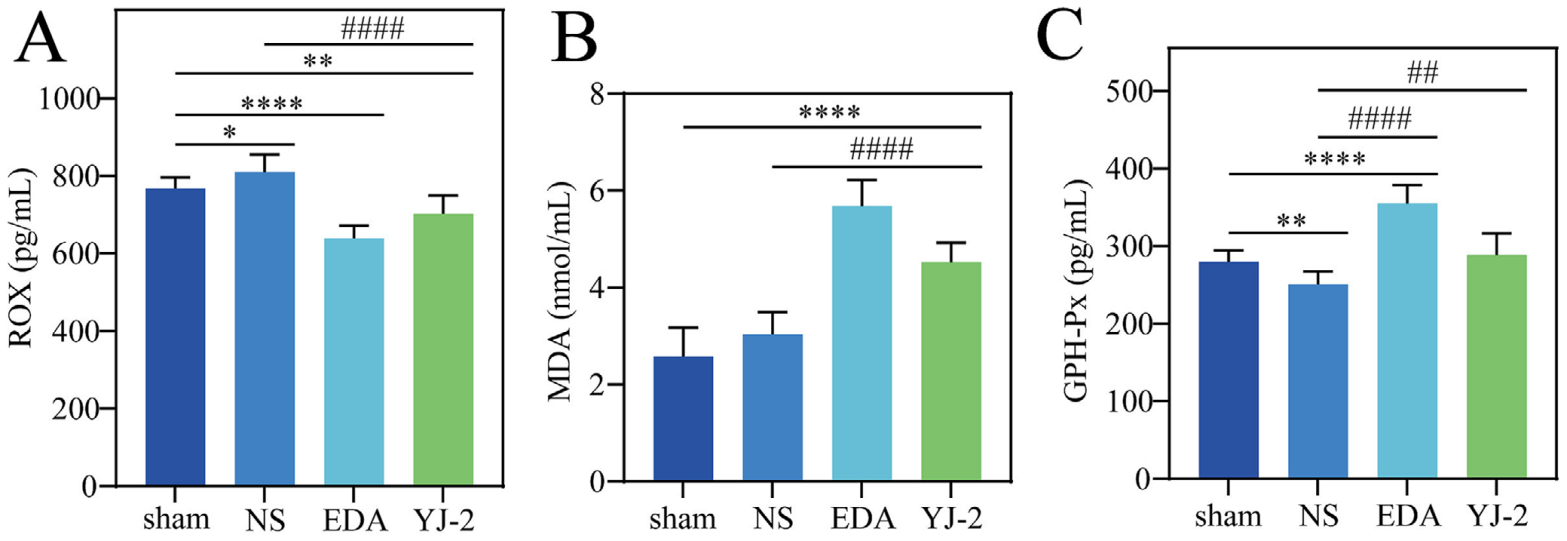
ELISA analysis of serum oxidative stress markers. Serum levels of (A) reactive oxygen species (ROS), (B) malondialdehyde (MDA), and (C) glutathione peroxidase (GSH‐Px) activity were determined (*n* = 6). Data are expressed as mean ± SD. *p < 0.05, **p < 0.01, ****p < 0.0001 versus Sham group. ^##^ *p* < 0.01, ^####^ *p* < 0.0001 versus NS group.

To evaluate the effect of YJ‐2 on systemic oxidative stress levels after cerebral ischemia–reperfusion, serum was collected from rats in each group at 22 h post‐reperfusion, and relevant markers were measured by ELISA. The ELISA results (Figure 4) showed that compared to the Sham group, the model (NS) group exhibited a significant increase in serum ROS levels and a marked decrease in the activity of the core antioxidant enzyme GSH‐Px, confirming successful activation of oxidative stress. YJ‐2 treatment demonstrated a differential regulatory effect: compared to the NS group, YJ‐2 effectively reduced ROS levels and enhanced GSH‐Px activity, indicating its antioxidant capacity. However, unexpectedly, YJ‐2 further significantly increased the content of MDA, a terminal product of lipid peroxidation. This seemingly paradoxical result suggests that YJ‐2 might directly or indirectly promote the process of lipid peroxidation through a specific mechanism. Nevertheless, its concurrent effects of reducing ROS and elevating GSH‐Px activity indicate that the neuroprotective effect of YJ‐2 might be partially independent of the classic MDA‐associated lipid peroxidation pathway, or achieved by scavenging other types of free radicals.

### 3.7 YJ‐2 Alleviates Neuronal Structural Damage After Cerebral Ischemia–Reperfusion

**FIGURE 5:**
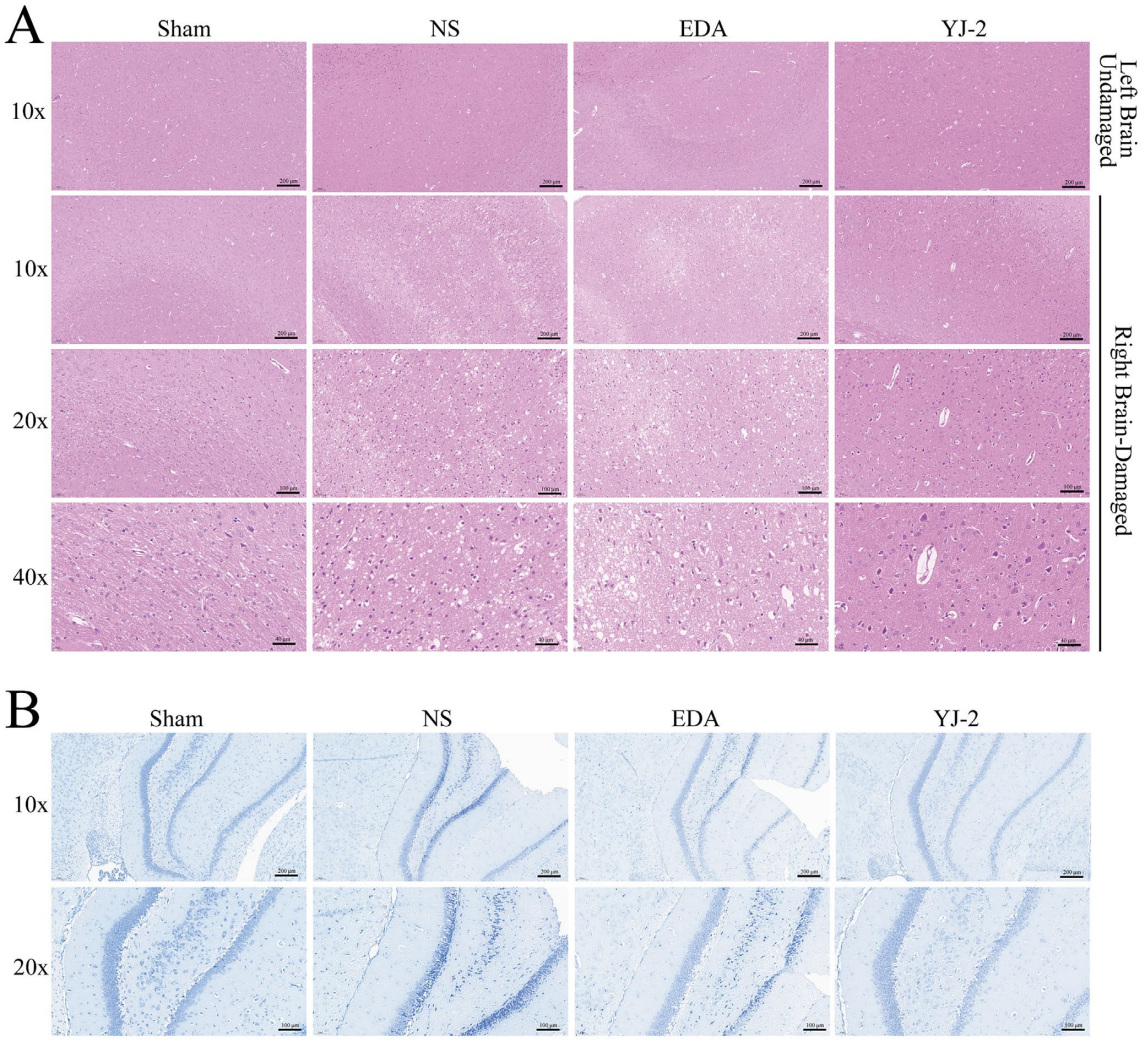
YJ‐2 alleviates neuronal structural damage after cerebral ischemia–reperfusion. (A) Representative H&E‐stained sections showing morphological changes in the cortical neurons at 22 h after reperfusion. (B) Nissl staining assessing the structural integrity of hippocampal neurons at 22 h after reperfusion.

To evaluate the neuroprotective effects of YJ‐2 at the histomorphological level, rat brain sections were analyzed using H&E and Nissl staining. H&E staining results (Figure 5A) showed that cortical neurons in the Sham group exhibited intact morphology, clear nuclei, and distinct nucleoli. In stark contrast, the model (NS) group displayed severe neuronal damage at 22 h post‐reperfusion, characterized by reduced neuronal numbers and typical ischemic changes such as nuclear pyknosis, hyperchromasia, and cytoplasmic shrinkage in the remaining cells. However, YJ‐2 treatment significantly ameliorated these pathological alterations. Compared to the NS group, the YJ‐2 treated group showed an increased number of neurons with intact structure in the cortical region and a significant reduction in the proportion of damaged neurons exhibiting pyknotic and hyperchromatic nuclei, indicating that YJ‐2 effectively mitigated I/R‐induced neuronal degeneration and necrosis.

Nissl staining results (Figure 5B) further corroborated these findings. Neurons in the hippocampal region of the Sham group displayed abundant Nissl bodies with plump morphology, whereas the NS group showed a significant reduction in Nissl body numbers, sparse distribution, and faint staining, suggesting severely impaired protein synthesis function. YJ‐2 intervention markedly restored the number of Nissl bodies in hippocampal neurons, resulting in more regular morphology and deeper staining. This indicates that YJ‐2 helps maintain normal neuronal synthetic metabolism, thereby preserving structural integrity.

In summary, histomorphological observations at the cellular level confirm that YJ‐2 effectively counteracts cerebral ischemia–reperfusion injury, alleviates structural damage to neurons, and helps maintain their functional activity.

### 3.8 YJ‐2 Suppresses the Expression of PAD4 and H3cit After Cerebral Ischemia–Reperfusion

**FIGURE 6:**
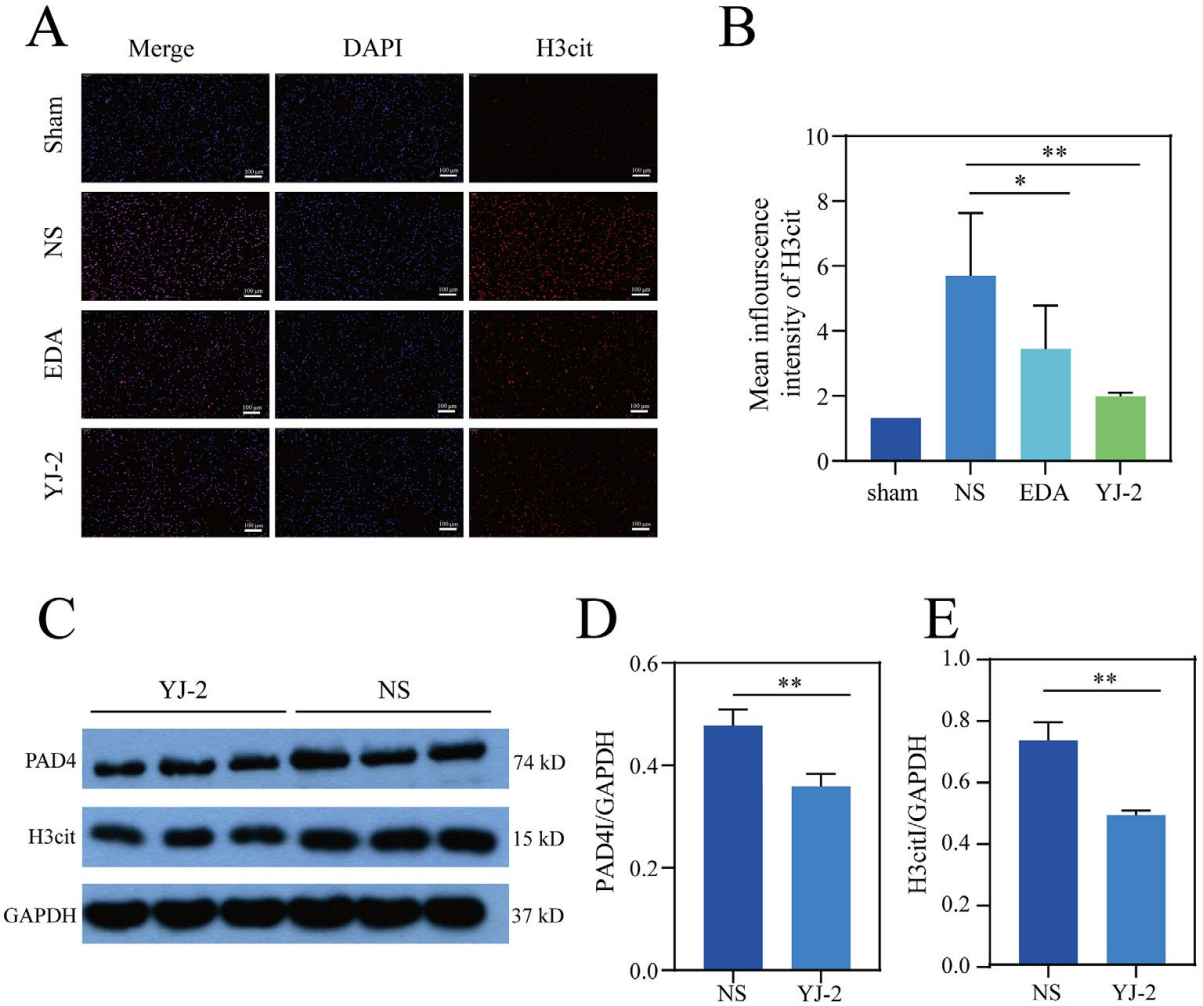
YJ‐2 suppresses the expression of PAD4 and H3cit after cerebral ischemia–reperfusion. (A) Representative immunofluorescence images of H3cit (red) in the ischemic cortex of Sham, NS, EDA, and YJ‐2 treated rats. Nuclei were counterstained with DAPI (blue) (scale bar: 100 μm). (B) Quantitative analysis of H3cit fluorescence intensity (*n* = 3). (C) Western blot analysis of PAD4 and H3cit protein bands. (D, E) Quantitative analysis of protein expression levels based on grayscale values (*n* = 3). Data are expressed as mean ± SD. *p < 0.05, **p < 0.01.

Studies have indicated that in the infarcted cortex following ischemia–reperfusion, neutrophils form NETs within the brain parenchyma and perivascular areas, thereby exacerbating neuroinflammation and tissue damage [30]. To validate the effect of YJ‐2 on the key molecular pathway of NETs formation, we detected the expression of PAD4 and its mediated H3cit via Western blot and immunofluorescence, respectively. The results showed that compared to the NS group, the protein levels of both PAD4 and H3cit in the brain were significantly reduced in the YJ‐2 treatment group (Figure 6C–E). Immunofluorescence analysis further confirmed a marked attenuation of H3cit‐positive signals in the YJ‐2 treated group (Figure 6A,B). Collectively, these results demonstrate that YJ‐2 effectively inhibits the activation of PAD4 and the subsequent histone citrullination process after cerebral ischemia–reperfusion, thereby interfering with NETs formation and providing molecular‐level insight into part of its neuroprotective mechanism.

### 3.9 BBB Penetration of YJ‐2 After Ischemic Injury

To determine whether YJ‐2 can cross the BBB following ischemic injury, brain tissues were collected from rats 3 h after the administration of YJ‐2 and subjected to Fourier Transform Mass Spectrometry (FT‐MS) analysis (for detailed sample preparation, see Supporting Information Methods). As shown in Figure S2, YJ‐2 was unequivocally detected in the brain homogenates of MCAO rats that received YJ‐2 injection. Critically, no corresponding signal for YJ‐2 was observed in the brains of rats from the NS‐treated control group (Figure S3). This result provides direct evidence that YJ‐2 is capable of penetrating the BBB under ischemic conditions, and the detected signal is specific to the administered compound.

## 4 Discussion

Ischemic stroke remains a devastating condition with limited therapeutic options beyond reperfusion. The intricate inflammatory cascade, particularly involving neutrophils, plays a pivotal role in exacerbating secondary brain injury following ischemia–reperfusion [30, 31, 32]. Our study identifies the PAD4‐NETs axis as a critical therapeutic target and demonstrates that its inhibition by YJ‐2 confers robust neuroprotection through a multi‐faceted mechanism.

The formation of NETs, a process known as NETosis, has emerged as a key driver of pathology in sterile inflammation, including ischemic stroke [33, 34]. Our data consistently show that YJ‐2, a novel PAD4 inhibitor, effectively suppresses this pathway. In vitro, YJ‐2 directly inhibited PMA‐induced NETosis (as inferred from the protective effects of its pretreated supernatant), and in vivo, it significantly reduced the levels of both PAD4 and its specific product, H3cit, in the ischemic brain (Figure 6). This places YJ‐2 among a promising class of therapeutic agents targeting PAD4 [24, 35]. With an IC_50_ of 0.836 μM against PAD4, YJ‐2 is approximately 7‐fold more potent than Cl‐amidine (IC_50_ = 5.9 μM) and also more potent than BB‐Cl‐amidine (IC_50_ = 1.1 μM), a gain driven by its optimized benzylamino and 4‐hydroxybenzamide groups [22, 26]. Notably, unlike Cl‐amidine and BB‐Cl‐amidine, which have required nanocarrier‐based strategies for brain delivery [24, 25], YJ‐2 penetrates the disrupted BBB and directly suppresses PAD4 activity and H3cit generation in ischemic tissue (Figures S2 and S3, Figure 6). The significance of our findings lies in the demonstration that this molecular‐level inhibition translates into a comprehensive protective phenotype across cellular and whole‐organism models.

A key strength of our study is the demonstration of YJ‐2's efficacy at multiple levels of the neurovascular unit. We confirmed that NETs components are directly toxic to neurons and profoundly disruptive to BBB integrity, consistent with prior reports [24, 36]. The novelty of our work lies in showing that a single agent, YJ‐2, can simultaneously mitigate both types of damage. The protection of the BBB in vitro (Figure 1C) is particularly crucial, as BBB disruption is a critical event that allows further infiltration of damaging immune cells into the brain parenchyma [37, 38]. The in vivo results powerfully validated these findings. The reduction in infarct volume (Figure 3A,B), coupled with the preservation of neuronal structure (Figure 5) and the sharp decrease in apoptosis (Figure 3D,E), provides a compelling histopathological basis for the observed improvement in neurological function (Figure 3C). This functional recovery is the ultimate goal of any stroke therapy, and YJ‐2's positive impact on the mNSS score underscores its translational potential.

An intriguing finding was the dissociation of oxidative stress markers: YJ‐2 reduced ROS but elevated serum MDA, a classic marker of lipid peroxidation (Figure 4). This paradox suggests that its antioxidant mechanism is complex. We postulate several non‐mutually exclusive hypotheses to explain this phenomenon:

Mechanistic Decoupling: The potent anti‐inflammatory and anti‐apoptotic effects of YJ‐2, achieved through PAD4/NETs inhibition, may constitute the dominant neuroprotective mechanism. The benefits derived from this primary action could simply outweigh the potential negative effects of elevated MDA, which might be a secondary or peripheral phenomenon. Shift in Cell Death Pathways: By strongly suppressing apoptosis (as shown by TUNEL), YJ‐2 might inadvertently shift the balance of cellular stress, potentially unmasking or mildly potentiating other forms of regulated cell death, such as ferroptosis, which is characterized by iron‐dependent lipid peroxidation [39]. Future studies measuring ferroptosis markers (e.g., GPX4, ACSL4) could investigate this potential crosstalk. This unexpected result does not diminish the value of YJ‐2 but rather highlights the complexity of its pharmacological profile and opens exciting new avenues for investigating its precise mechanism of action.

While our study provides compelling evidence, several limitations should be acknowledged. Firstly, although we demonstrated that YJ‐2 inhibits the PAD4/H3cit/NETosis axis, future studies utilizing cell‐specific PAD4 knockout models would more directly ascertain the cellular target of YJ‐2 in vivo. Secondly, as discussed above, the precise mechanism behind the elevated MDA warrants further investigation into the compound's specific effects on lipid metabolism and other cell death pathways. Lastly, the long‐term benefits and the critical therapeutic time window for YJ‐2 administration remain to be elucidated in extended studies. Exploring its combination with thrombolytic therapy (e.g., t‐PA) also represents a critical next step for clinical translation.

## 5 Conclusions

In summary, this study establishes a comprehensive evidential chain spanning from molecular and cellular levels to whole‐organism models: YJ‐2, via specific inhibition of PAD4, suppresses NETs formation, thereby alleviating their direct neurotoxicity, preserving BBB integrity, and ultimately achieving effective defense against ischemic brain damage in vivo by inhibiting apoptosis and preserving neuronal structure and function. This work not only validates YJ‐2 as a promising candidate therapeutic agent but also further solidifies the scientific rationale for targeting the PAD4/NETs pathway in the treatment of ischemic stroke. Future research will focus on exploring the potential of combining YJ‐2 with other treatment strategies (such as thrombolysis) and investigating the downstream signaling mechanisms through which it exerts its neuroprotective effects.

## Author Contributions

**Yijiang Jia:** methodology, validation, writing – original draft, project administration, resources. **Ayijiang Taledaohan:** methodology, validation, writing – original draft, visualization. **Kai Wang:** formal analysis, investigation, methodology, visualization. **Maer Maer Tuohan:** data curation, formal analysis. **Rong Chen:** data curation, validation. **Liujia Chan:** methodology, validation. **Haoyu Shen:** investigation, methodology. **Di Zhu:** funding acquisition, supervision, writing – review and editing. **Yuji Wang:** conceptualization, funding acquisition, project administration, supervision, writing – review and editing. All authors have read and agreed to the published version of the manuscript.

## Funding

This study was supported by the Beijing High‐Level Innovative and Entrepreneurial Talent Support Program “Capital High‐End Leading Talent Gathering and Cultivation Project” (202504841074), National Natural Science Foundation of China for Young Scholars (No. 22407094), and the Natural Science Foundation of Beijing Municipality for Young Scholars (No. 2254070).

## Ethics Statement

All experimental procedures were approved by the Institutional Animal Care and Use Committee of Capital Medical University (Ethics Approval Number: AEEI‐2020‐188) and conducted in accordance with the Regulations on Laboratory Animal Welfare issued by the Chinese Ministry of Science and Technology.

## Conflicts of Interest

The authors declare no conflicts of interest.

## Supporting information


**Figure S1:** Chemical structure of compound YJ‐2 (exact mass: 416.1615, C_21_H_25_ClN_4_O_3_).
**Figure S2:** Full‐scan high‐resolution mass spectrum of compound YJ‐2 in mouse brain homogenate post‐administration.
**Figure S3:** Full‐scan high‐resolution mass spectrum of brain homogenate from the NS control group, confirming the absence of compound YJ‐2.

## Data Availability

The data that support the findings of this study are available from the corresponding author upon reasonable request.
