# Supplementary material for: Comprehensive Evaluation of YJ‐2 as a PAD4 Inhibitor in Alleviating Ischemic Brain Injury: From NETs‐Induced Neurotoxicity to In Vivo Neuroprotection
Source: CNS Neurosci Ther. 2026 Jul 15;32(7):e71032. doi: 10.1002/cns.71032 (PMC13371606; doi:10.1002/cns.71032)
Supplement: Supplementary file 1 — Figure S1: Chemical structure of compound YJ‐2 (exact mass: 416.1615, C21H25ClN4O3). Figure S2: Full‐scan high‐resolution mass spectrum of compound YJ‐2 in mouse brain homogenate post‐administration. Figure S3: Full‐scan high‐resolution mass spectrum of brain homogenate from the NS control group, confirming the absence of compound YJ‐2. [file CNS-32-e71032-s001.docx]

**Supplementary Material**

**Comprehensive Evaluation of YJ-2 as a PAD4 Inhibitor in Alleviating Ischemic Brain Injury: From NETs-Induced Neurotoxicity to *In Vivo* Neuroprotection**

Yijiang Jia^1,2^, Ayijiang Taledaohan^3^, Kai Wang^1,2^, Maer Maer Tuohan^1,2^, Rong Chen^4^, Liujia Chan^1,2^, Haoyu Shen^1,2^, Di Zhu^5^, Yuji Wang^1,2^*

^1^ Department of Medicinal Chemistry, School of Pharmaceutical Sciences of Capital Medical University, 10 Xi Tou Tiao, You An Men, Beijing 100069, PR China.

^2^ Beijing Key Laboratory of Drug Innovation for Neuro-Oncology, Beijing Engineering Research Center of Targeted Drugs and Cell Therapy for CNS Tumors, Laboratory for Clinical Medicine, Capital Medical University, Beijing 100069, PR China.

^3^ Department of Emergency Medicine at the University of California San Diego, San Diego, CA 92103, United States.

^4^ Department of Clinical pharmacy,The First Affiliated Hospital of Shandong First Medical University & Shandong Provincial Qianfoshan Hospital, Jinan, Shandong 250014, PR China.

^5^ Department of Pharmacy, Xuanwu Hospital, Capital Medical University, Beijing 100053, PR China.

*Corresponding authors:

E-mail address: [wangyuji@ccmu.edu.cn](mailto:wangyuji@ccmu.edu.cn(Y) (Y. Wang).

**Sample Preparation for Fourier Transform Mass Spectrometry (FT-MS)**

Brain tissues were collected from MCAO rats at 3 h post-administration of YJ-2. For FT-MS analysis, samples were prepared as follows: each tissue sample was homogenized in 200 μL of ice-cold phosphate-buffered saline (PBS) with four strokes. A 100 μL aliquot of the homogenate was then mixed with 300 μL of HPLC-grade methanol and 300 μL of HPLC-grade acetonitrile. The mixture was sonicated for 5 minutes and centrifuged at 19,083 × g for 10 minutes at 4°C to precipitate proteins. Subsequently, 600 μL of the supernatant was transferred and concentrated to dryness using a vacuum centrifugal concentrator at 50°C for 3.5 h. The resulting residue was reconstituted in HPLC-grade methanol, filtered through a microporous membrane, and transferred to a clean vial for FT-MS analysis. This sample preparation procedure is a standard laboratory protocol routinely established in our group, and a similar procedure was performed according to our previously described method.^[1, 2]^





Figure S1 Chemical structure of compound YJ-2 (Exact Mass:416.1615, C_21_H_25_ClN_4_O_3_).


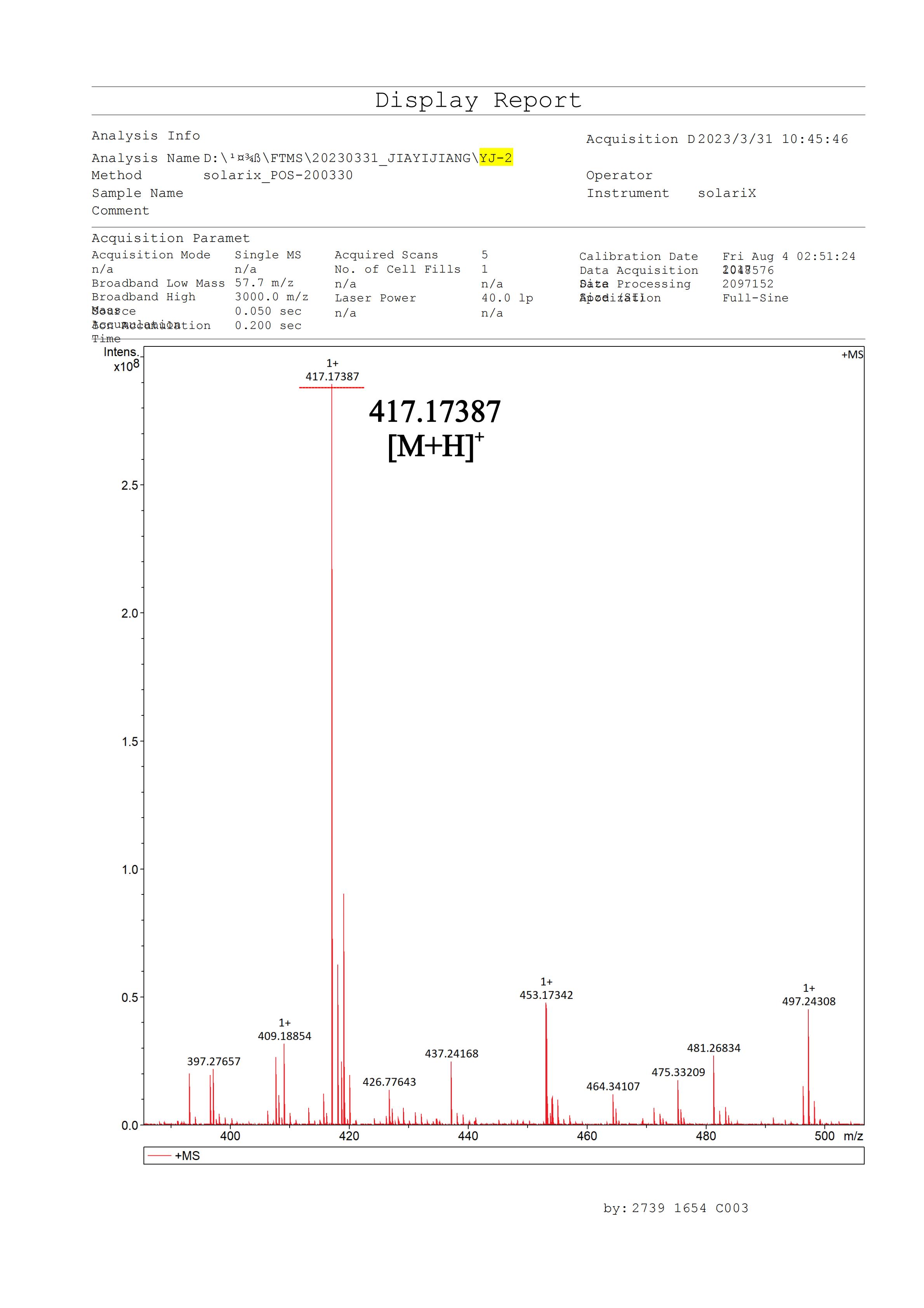


Figure S2 Full-scan high-resolution mass spectrum of compound YJ-2 in mouse brain homogenate post-administration.


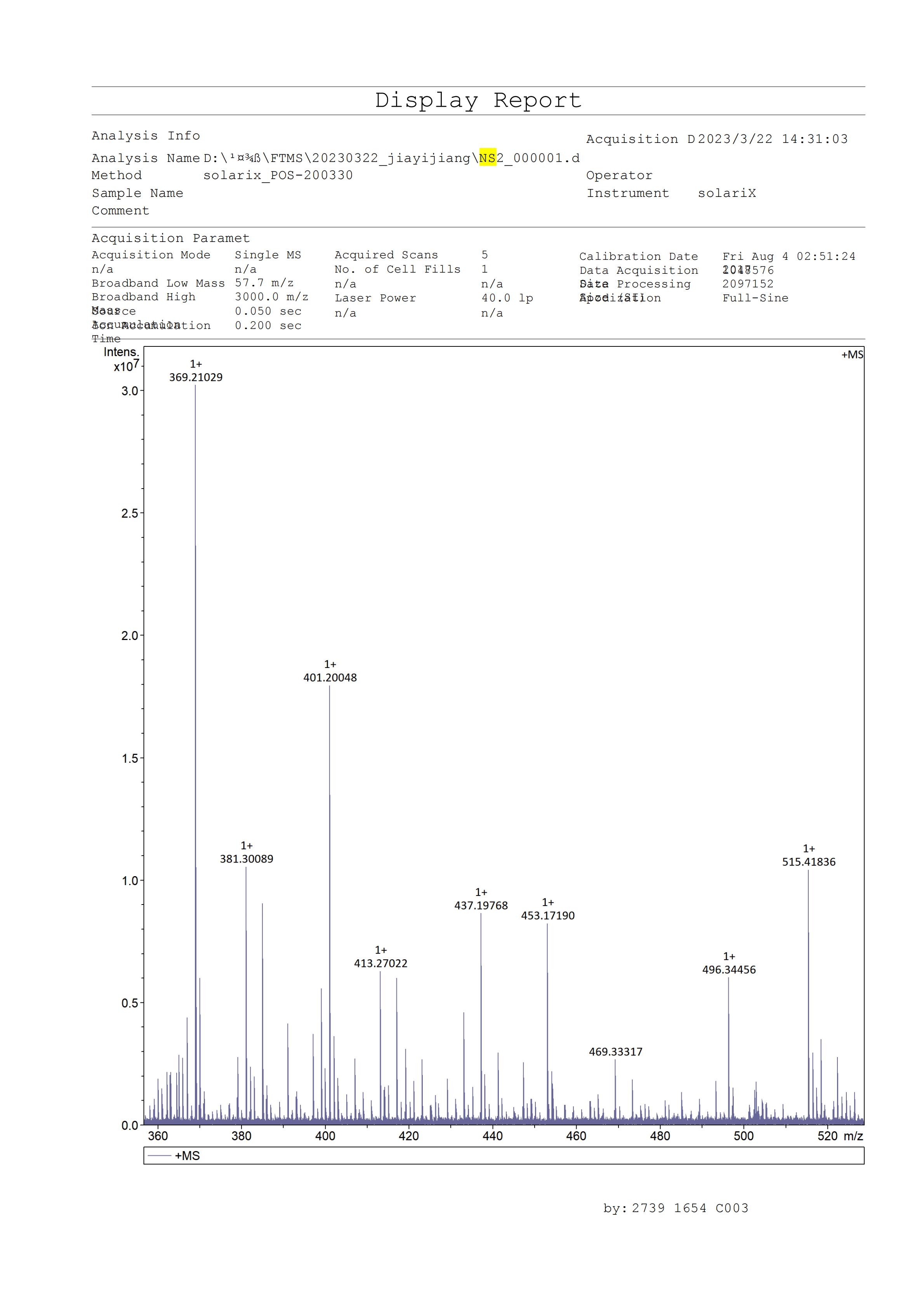


Figure S3 Full-scan high-resolution mass spectrum of brain homogenate from the NS control group, confirming the absence of compound YJ-2.

**Reference**

[1] Jia, Y., Taledaohan, A., Jia, R., Wang, X., Jia, Y., Liu, J., Wang, Y. Chitosan nanomedicine containing RGD peptide and PAD4 inhibitor based on phenyl boronate coupling inhibition of primary tumor growth and lung metastasis. *Biomed Pharmacother*, 2023, 168: 115826.

[2] Taledaohan, A., Tuohan, M. M., Jia, R., Wang, K., Chan, L., Jia, Y., Wang, F., Wang, Y. An RGD-Conjugated Prodrug Nanoparticle with Blood-Brain-Barrier Penetrability for Neuroprotection Against Cerebral Ischemia-Reperfusion Injury. *Antioxidants (Basel)*, 2024, 13(11).
